# The *Pythium periplocum* elicitin PpEli2 confers broad-spectrum disease resistance by triggering a novel receptor-dependent immune pathway in plants

**DOI:** 10.1093/hr/uhac255

**Published:** 2022-11-15

**Authors:** Kun Yang, Yi Wang, Jialu Li, Yaxin Du, Ying Zhai, Dong Liang, Danyu Shen, Rui Ji, Xuexiang Ren, Hao Peng, Maofeng Jing, Daolong Dou

**Affiliations:** Key Laboratory of Biological Interaction and Crop Health, Department of Plant Pathology, Nanjing Agricultural University, Nanjing 210095, China; Key Laboratory of Biological Interaction and Crop Health, Department of Plant Pathology, Nanjing Agricultural University, Nanjing 210095, China; Key Laboratory of Biological Interaction and Crop Health, Department of Plant Pathology, Nanjing Agricultural University, Nanjing 210095, China; Key Laboratory of Biological Interaction and Crop Health, Department of Plant Pathology, Nanjing Agricultural University, Nanjing 210095, China; Department of Plant Pathology, Washington State University, Pullman, WA 99164, USA; Key Laboratory of Biological Interaction and Crop Health, Department of Plant Pathology, Nanjing Agricultural University, Nanjing 210095, China; Key Laboratory of Biological Interaction and Crop Health, Department of Plant Pathology, Nanjing Agricultural University, Nanjing 210095, China; Institute of Plant Protection, Jiangsu Academy of Agricultural Sciences, Nanjing 210014, China; Institute of Plant Protection and Agro-Products Safety, Anhui Academy of Agricultural Sciences, Hefei 230001, China; Department of Plant Pathology, Washington State University, Pullman, WA 99164, USA; Key Laboratory of Biological Interaction and Crop Health, Department of Plant Pathology, Nanjing Agricultural University, Nanjing 210095, China; Key Laboratory of Biological Interaction and Crop Health, Department of Plant Pathology, Nanjing Agricultural University, Nanjing 210095, China; Academy for Advanced Interdisciplinary Studies, Nanjing Agricultural University, Nanjing 210095, China

## Abstract

Elicitins are microbe-associated molecular patterns produced by oomycetes to elicit plant defense. It is still unclear whether elicitins derived from non-pathogenic oomycetes can be used as bioactive molecules for disease control. Here, for the first time we identify and characterize an elicitin named PpEli2 from the soil-borne oomycete *Pythium periplocum*, which is a non-pathogenic mycoparasite colonizing the root ecosystem of diverse plant species. Perceived by a novel cell surface receptor-like protein, REli, that is conserved in various plants (e.g. tomato, pepper, soybean), PpEli2 can induce hypersensitive response cell death and an immunity response in *Nicotiana benthamiana*. Meanwhile, PpEli2 enhances the interaction between REli and its co-receptor BAK1. The receptor-dependent immune response triggered by PpEli2 is able to protect various plant species against *Phytophthora* and fungal infections. Collectively, our work reveals the potential agricultural application of non-pathogenic elicitins and their receptors in conferring broad-spectrum resistance for plant protection.

## Introduction

*Phytophthora* is an oomycete genus causing some of the most notorious plant diseases in the world [1–4]. For example, *Phytophthora capsici* is a highly dynamic and destructive pathogen, which attacks important vegetables such as pepper, tomato, eggplant, and cucurbits [5]. *Phytophthora parasitica* causes the notable tobacco diseases of brown rot, foot rot, and black shank, which affect almost all tobacco-growing areas worldwide, with losses as high as 100% [6]. *Phytophthora sojae* infection leads to soybean root rot, causing an annual loss of $1–2 billion worldwide [7, 8]. Chemical pesticides and plant disease resistance (*R*) genes can be used to defend *Phytophthora* pathogens. However, *Phytophthora* pathogens evolve rapidly to overcome fungicides and *R* gene-mediated resistance, and this leads to a continuous demand for sustainable approaches to confer broad-spectrum resistance (BSR) against *Phytophthora* and other pathogens.

Development of novel *Phytophthora* control methods relies on the dissection of the plant immune system [9]. Plants use pattern-recognition receptors (PRRs) at the cell surface to perceive evolutionarily conserved microbe- or pathogen-associated molecular patterns (MAMPs or PAMPs) [10, 11], and thereby trigger immunity (MAMP- or PAMP-triggered immunity, MTI or PTI) [12, 13]. MTI/PTI is characterized by multiple hallmarks, including callose deposition, reactive oxygen species (ROS) burst, defense-related gene expression, and activation of mitogen-activated protein kinases (MAPKs) [14, 15]. Plants also recognize microbial avirulence effectors, usually through intracellular nucleotide-binding leucine-rich repeat (LRR) receptors (NLRs), which are often encoded by *R* genes, to trigger immunity (effector-triggered immunity, ETI) [16]. ETI is also able to potentiate MTI/PTI to defend the plant against pathogens [16–18].

PRRs are usually plasma membrane-associated LRR receptor-like kinases (RLKs) or proteins (RLPs) [19–21]. Both LRR-RLKs and LRR-RLPs contain an extracellular domain and a transmembrane domain, but LRR-RLPs lack the cytoplasmic kinase domain [19, 22]. PRRs are capable of recognizing MAMPs as well as damage-associated molecular patterns (DAMPs) [9, 23]. For example, *Arabidopsis thaliana* utilizes LRR-RLKs FLS2 and EFR to perceive bacterial peptides flg22 and elf18, respectively [24–26]. Fungal ethylene-inducing xylanase (EIX), *Phytophthora infestans* elicitin INF1, *P. sojae* XEG1, and Nep1-like proteins (NLPs) can all be recognized by their corresponding LRR-RLPs [9, 27–34]. Some PRRs function in immune signaling as co-receptors, including two highly conserved LRR-RLKs, BRI1-ASSOCIATED KINASE 1 (BAK1) and SUPPRESSOR OF BAK1-INTERACTING RECEPTOR-LIKE KINASE 1 (SOBIR1) [16, 35]. BAK1 is involved in both RLK and RLP signaling [36], while SOBIR1 mainly regulates RLP-mediated signal transmission [35]. Some PRRs are characterized only in a narrow range of plant species. For example, EFR exists exclusively in Brassicaceae species. ELICITIN RESPONSE (ELR) is identified specifically from *Solanum microdontum* genotype mcd360-1 [29]. Ectopic expression of EFR in tomato and *Nicotiana benthamiana* confers BSR against bacteria despite plant species or genotype specificity [37]. Introduction of ELR into cultivated potato varieties enhances their resistance to *P. infestans*. These observations suggest that PRRs and the PAMPs/MAMPs they recognize are valuable resources for developing disease-resistant crops.

In recent decades, crop disease control has mainly depended on the use of chemical pesticides. However, several pathogens have evolved resistance to frequently used pesticides [38]. In addition, some pesticides are not readily broken down into simple and safer constituents, and eventually exist as toxic residues in the soil [39]. Increasing public concerns on environmental and health issues associated with synthetic chemicals are causing a shift towards more sustainable and eco-friendly disease management practices [38, 40]. Given this situation, a large number of biological control agents have been developed and applied [41]. PRRs and MAMPs are potential biocontrol agents for conferring BSR against oomycete and fungal pathogens.

MAMPs from non-pathogenic microbes are of particular interest for biocontrol. The mycoparasite *Pythium periplocum* and its sister species *Pythium oligandrum* are non-pathogenic oomycetes that colonize the plant root ecosystem of a large number of plant species [42]. Distinguished from pathogenic oomycetes, *P. oligandrum* triggers plant immune responses through its MAMPs, and produces the auxin precursor tryptamine to promote plant growth. *P. oligandrum* has been successfully used as a biocontrol agent for controlling various plant diseases, including those caused by *Phytophthora* pathogens [42–44]. MAMPs identified in *P. oligandrum* include POD-1/2, NLPs, and oligandrins (Oli-D1 and Oli-D2) classified as elicitins [45–47]. Elicitins are structurally conserved extracellular proteins found in *Phytophthora* and *Pythium* species. Their encoding genes occur as complex multigene families and form diverse subclasses of elicitin and elicitin-like genes [48]. All elicitins share a highly conserved 98-amino-acid (aa) domain with six cysteine residues forming three disulfide bridges. Elicitin-like proteins also have six structurally conserved cysteine residues, but the lengths of their elicitin domains and cysteine-spacing patterns vary within each phylogenetic clade member [48, 49]. Elicitins are able to trigger hypersensitive response (HR) cell death, ROS burst, and disease resistance in several plants [48]. LRR-RLP ELR from *S. microdontum* has been identified as a PRR to recognize the *P. infestans* elicitin INF1 [29]. However, elicitin PRRs in other plant species are largely unknown.

Here, we identified a novel elicitin-like PpEli2 from *P. periplocum*. PpEli2 induces HR and ROS burst, which enhance plant BSR to multiple *Phytophthora* and fungal pathogens*.* We further demonstrated that an *N. benthamiana* LRR-RLP mediates the perception of PpEli2, and named it REli (Receptor of PpEli2). REli is associated with NbBAK1 and NbSOBIR1. PpEli2 enhances the interaction between REli and BAK1 *in planta*. Ectopic expression of PpEli2 is able to enhance BSR against various *Phytophthora* and fungal pathogens in tomato, pepper, and soybean. Our work demonstrates that PpEli2 is a novel MAMP with promising biocontrol applications.

## Results

### PpEli2 induces cell death and plant resistance in *N. benthamiana*

**Figure 1:**
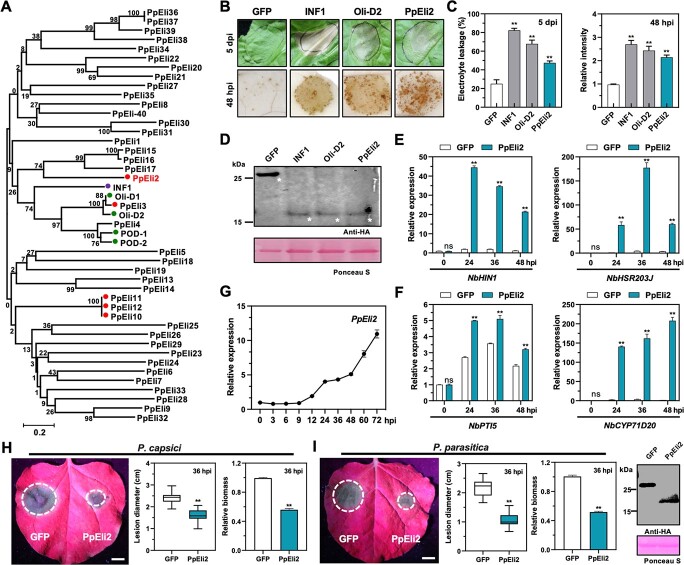
The elicitin-like PpEli2 induces HR cell death and plant resistance in *N. benthamiana*. (A) Phylogenetic tree of elicitins and elicitin-like proteins from *P. periplocum*. Green and purple circles indicate elicitins reported to induce cell death or ROS. Red circles indicate elicitins identified in this research as inducers of cell death or ROS. Oli-D1, Oli-D2, POD-1, and POD1-2 are elicitin-like proteins identified from *P. oligandrum*. INF1 is an elicitin from *P. infestans*. (B) Transient expression of GFP, INF1, Oli-D2, and PpEli2 individually in *N. benthamiana* by agroinfiltration. Photographs of cell death (upper panel) and H_2_O_2_ accumulation (lower panel) phenotypes were taken at 5 days post-infiltration (dpi) and 48 hpi, respectively. INF1 and Oli­D2 served as positive controls and GFP as negative control, respectively. (C) Quantifications of electrolyte leakage induced by cell death and relative intensities of H_2_O_2_ accumulation. All treatment groups are described in (B). Error bars represent mean ± standard deviation (*n* ≥ 9; *n* represents sample number). (D) Immunoblot detection of GFP, INF1, Oli-D2, and PpEli2 using anti-HA antibody. (E, F) Relative expression of HR (E) and MTI (F) marker genes in *N. benthamiana* leaves. Agroinfiltrated leaves were collected at the indicated time points to analyze the expression levels of *NbHIN1*, *NbHSR203J*, *NbPTI5*, and *NbCYP71D20* by qRT–PCR. GFP was used as a negative control. Error bars represent mean ± standard deviation (*n* ≥ 8). (G) Time-series expression profile of *PpEli2* after leaf inoculation with *P. periplocum*. Total RNAs were extracted from *P. periplocum* mycelia (0 hpi) or inoculated *N. benthamiana* leaves at 3–72 hpi. Transcript accumulation levels of *PpEli2* during plant–*P. periplocum* interaction was determined by qRT–PCR. The *P. periplocum Actin* gene was used as the housekeeping gene. Error bars represent mean ± standard deviation (*n* = 8; *n* represents sample number). (H, I) PpEli2 enhanced plant resistance of *N. benthamiana* leaves to *P. capsici* (H) and *P. parasitica* (I) infections. Scale bar = 1 cm. Relative biomass was determined by qPCR using references *P. capsici* or *P. parasitica Actin* and *N. benthamiana EF1α*. A representative immunoblot shows the protein levels of transiently expressed GFP and PpEli2 *in planta*. Error bars represent mean ± standard deviation (*n* ≥ 10). Data were analyzed by the Shapiro–Wilk test to determine normality and log normality across various groups. Groups passing the normality test were then analyzed by the unpaired *t* test (^**^, *P* < .01). These experiments were performed with least three biological replicates, with similar results.

To identify putative elicitins in *P. periplocum*, we used a hidden Markov model of the conserved elicitin domain (Pfam PF00964) to search the proteome of *P. periplocum* strain CBS 532.74. Meanwhile, the SignalP v3.0 program was used to identify the N-terminal signal peptides. In total, 40 elicitin-like genes, but no elicitin gene, were found in the *P. periplocum* genome. The encoded proteins were named PpEli1–40, with none of their elicitin domains being the typical 98 aa in length (Supplementary Data Table S1). All 40 *PpEli* genes were cloned and then transiently expressed in leaves of *N. benthamiana* for cell death screening. Five PpElis were found to induce cell death in *N. benthamiana* (Supplementary Data Fig. S1). Among them, *PpEli3* is a homolog of *Oli-D1/D2* from *P. oligandrum*. *PpEli10–12* encode identical aa sequences. PpEli2, PpEli3, and PpEli10–12 are evolutionarily separated (Fig. 1A).

PpEli2 was chosen for further investigation due to its strong cell death- and ROS-inducing activity (Fig. 1B–D). In relevant assays, we used two elicitins, *P. infestans* INF1 [50] and *P. oligandrum* Oli-D2, as two positive controls, and GFP as a negative control. To evaluate plant immunity responses in *N. benthamiana*, we examined the expression levels of two cell death markers, *HSR203J* and *HARPIN-INDUCED 1* (*HIN1*) [46, 51, 52], as well as two PTI markers, *NbCYP71D20* and *NbPTI5* [9, 53]. Quantitative reverse transcription polymerase chain reaction (qRT–PCR) analysis showed that transient expression of PpEli2 significantly upregulated all four marker genes (Fig. 1E and F), indicating its strong immune response-inducing activity. Furthermore, *PpEli2* expression was induced during the interaction between *P. periplocum* and *N. benthamiana* (Fig. 1G). Ectopic expression of PpEli2 significantly reduced *P. capsici* colonization in *N. benthamiana*. We used *Agrobacterium tumefaciens*-mediated transient expression technology to express GFP and PpEli2 in *N. benthamiana*; then, the leaves were inoculated with *P. capsici* mycelia plugs at 24 h after infiltration; the lesion and *P. capsici* biomass were measured after 36 hours of wet culture at 25°C in the dark (Fig. 1H). Similarly, PpEli2 also conferred resistance of *N. benthamiana* to *P. parasitica* (Fig. 1I). Together, the results demonstrate that PpEli2 is able to enhance resistance against various *Phytophthora* pathogens*.*

### PpEli2 triggers MAMP-triggered immunity and broad-spectrum resistance in *N. benthamiana*

**Figure 2:**
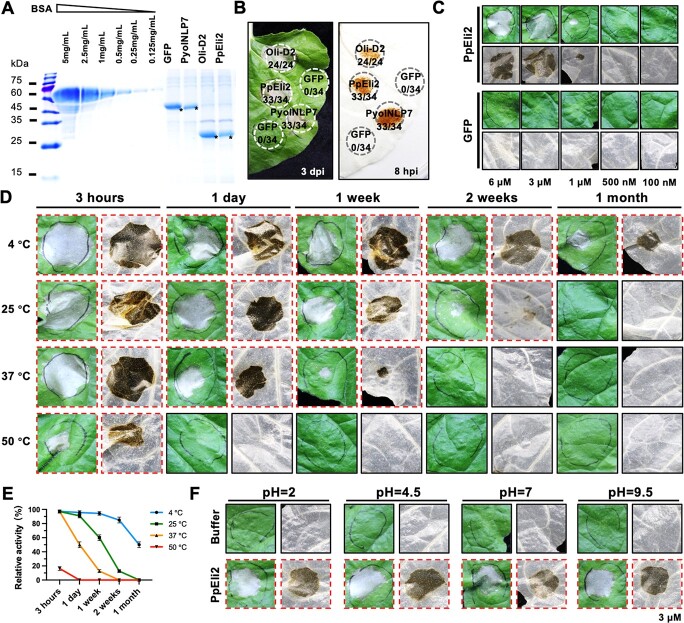
PpEli2-induced cell death and H_2_O_2_ accumulation in *N. benthamiana* under different heat and pH treatments. (A) Purified His-tagged PpEli2 and control proteins stained with Coomassie blue. ^*^, objective bands. (B) Cell death- and H_2_O_2_ accumulation-inducing activity of PpEli2 (3 μM). Oli-D2 and PyolNLP7 served as positive controls. For the HR assay, 3 μM protein was infiltrated into *N. benthamiana* leaves, and the HR phenotype was observed at 3 days post-infiltration (dpi). For the H_2_O_2_ accumulation assay, 3 μM protein was infiltrated into *N. benthamiana* leaves. At 8 hours hpi the leaf was stained with DAB solution for 10 hours. After alcohol decolorization, the phenotype as shown in the figure was observed. (C) Representative leaves of *N. benthamiana* infiltrated with purified 100 nM to 6 μM PpEli2 protein or GFP protein. Photographs were taken at 3 dpi. (D, E) Evaluation of PpEli2 thermal stability by examining changes in its cell death-inducing activity (D) and statistical quantifications (E). The thermal treatment range was 4–50°C. Treatments lasted from 3 h to 1 month. Error bars represent mean ± standard deviation (*n* = 9). (F) Effect of pH on PpEli2 stability as reflected by the cell death-inducting phenotype. These experiments were performed using at least four biological replicates with similar results.

**Figure 3:**
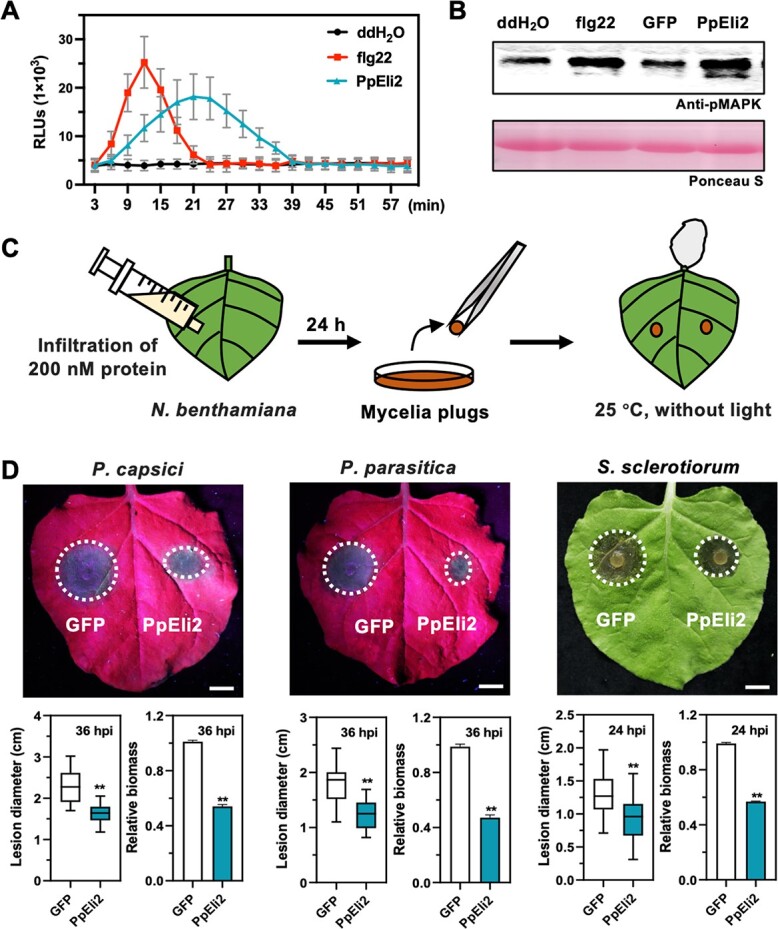
PpEli2 induces immune responses and increases *N. benthamiana* resistance to *Phytophthora* and fungal pathogens. (A) ROS production in *N. benthamiana* treated with 1 μM PpEli2. flg22 was used as a positive control and ddH_2_O as a negative control. Error bars represent mean ± standard deviation (*n* ≥ 9). (B) PpEli2 (1 μM) induced activation of MAPKs. flg22 was used as a positive control and ddH_2_O and GFP as negative controls. (C) Schematic diagrams of *N. benthamiana* leaves inoculated with pathogens after protein treatment. (D) Pretreatment with 200 nM PpEli2 protein induced plant resistance against *P. capsici*, *P. parasitica*, and *S. sclerotiorum*. In this assay, 200 nM GFP was used as control. Photos were taken at 36 and 24 hpi. Scale bar = 1 cm. Lesion diameters and relative biomass were measured at the indicated time points. Error bar represents mean ± standard deviation (*n* ≥ 9). Data were analyzed by the Shapiro–Wilk test to determine normality and log normality across groups. Groups passing the normality test were then analyzed by the unpaired *t* test (**, *P* < .01). These experiments were performed three times with similar results.

Purified recombinant PpEli2 protein produced from *Escherichia coli* strain BL21 (DE3) (Fig. 2A, Supplementary Data Table S2) significantly induced cell death and H_2_O_2_ accumulation in *N. benthamiana* leaves (Fig. 2B). Protein infiltration assays showed that the HR cell death-inducing ability of PpEli2 was positively correlated with protein concentration (Fig. 2C), negatively correlated with temperature, and decreased gradually over time that can be still induce HR saved after >1 month at 4°C and 2 weeks at 25°C (room temperature) (Fig. 2D and E). Meanwhile, we found PpEli2 was able to induce cell death and H_2_O_2_ accumulation under different pH values (Fig. 2F). In a luminol-based chemiluminescence assay, PpEli2 induced a rapid ROS burst in *N. benthamiana* leaves (Fig. 3A). Furthermore, immunoblotting using the phosphor-p44/42 MAPK antibody showed that, like the classic PAMP flg22, PpEli2 induced MAPK activation in *N. benthamiana* within 30 minutes (Fig. 3B). Both assays demonstrated that PpEli2 is able to trigger PTI in plants. To further examine PpEli2-mediated protection against oomycete and fungal pathogens, leaves of *N. benthamiana* were pretreated with either 200 nM PpEli2 or GFP 24 h before the inoculation of *P. capsici* mycelium plugs (Fig. 3C). Pretreatment with PpEli2 significantly reduced lesions and pathogen biomass compared with GFP (Fig. 3D). Similarly, PpEli2 enhanced *N. benthamiana* resistance against *P. parasitica* and *Sclerotinia sclerotiorum* (Fig. 3D). Together, these results suggest that PpEli2 protein is able to induce plant resistance rapidly and can potentially be used as a bioactive molecule.

### PpEli2-mediated immunity requires REli and co-receptors BAK1 and SOBIR1

**Figure 4:**
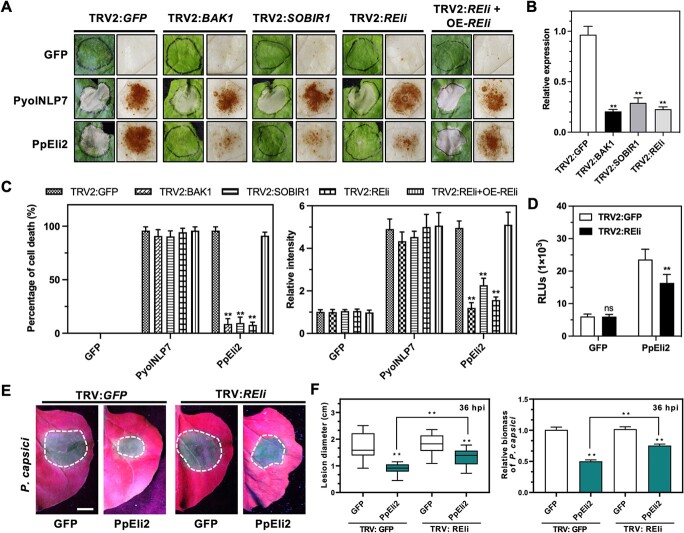
PpEli2-triggered immune response depends on a novel LRR-RLP REli and two co-receptors, BAK1 and SOBIR1. (A) PpEli2-induced HR (left) and H_2_O_2_ accumulation (right) require the LRR-RLP REli and its co-receptors BAK1 and SOBIR1. *REli*, *NbBAK1*, and *NbSOBIR1* were individually silenced by a TRV-based VIGS system. TRV-GFP was used as a control. TRV-REli+OE-REli indicates that REli is overexpressed in *REli-*silenced plants*.* 3 μM purified proteins were infiltrated into the *N. benthamiana* leaves. PyolNLP7 and GFP were used as positive and negative controls, respectively. Photographs were taken at 3 days post-infiltration (dpi) (left) and 8 hpi (right). (B) Expression levels of *REli*, *NbBAK1*, and *NbSOBIR1*. Error bars represent mean ± standard deviation (*n* ≥ 9). (C) Quantification of cell death percentages and relative intensities of H_2_O_2_ accumulation. Error bars represent mean ± standard deviation (*n* ≥ 10). (D) ROS production in *N. benthamiana* leaves treated with 1 μM PpEli2 recombinant proteins using a luminol-based chemiluminescence assay. Error bars represent mean ± standard deviation (*n* ≥ 8). (E) Silencing of *REli* attenuated PpEli2-mediated resistance to *P. capsic*i in *N. benthamiana*. *GFP* (mock)- or *REli*-silenced leaves were infiltrated with PpEli2 or GFP (200 nM), and then challenged with *P. capsici* for 48 hpi. (F) *P. capsici*-caused disease lesions and relative biomass of *P. capsici* were measured at the indicated time points. Error bars represent mean ± standard deviation (*n* ≥ 11). Data were analyzed by the Shapiro–Wilk test to determine normality and log normality across groups. Groups passing the normality test were then analyzed by one-way ANOVA with the *post hoc* Dunnett’s multiple comparisons test (^**^, *P* < .01; ns, no significant difference). All the experiments were performed with at least four biological replicates.

To identify PpEli2 perception PRRs, we screened LRR-RLK/RLP genes in *N. benthamiana* using a high-throughput *Tobacco rattle virus* (TRV)-induced gene silencing (VIGS) assay. The results showed that PpEli2-induced cell death was significantly impaired in an LRR-RLP silencing line (Niben101Scf02826g01005.1), and the corresponding protein was defined as REli (Fig. 4A–C, Supplementary Data Fig. S2). The PpEli2-induced ROS burst was also significantly weakened in *REli*-silenced plants using a luminol-based chemiluminescence assay (Fig. 4D). PpEli2-induced cell death was suppressed in *REli*-silenced plants but could be recovered by co-overexpressing the full-length *REli* gene as complementation (Fig. 4A–C, Supplementary Data Fig. S3). Furthermore, PpEli2-mediated resistance to *P. capsici* was impaired in *REli*-silenced *N. benthamiana* (Fig. 4E and F). Collectively, these results suggest that REli mediates a PpEli2-triggered immune response, likely by serving as its recognition receptor.

BAK1 and SOBIR1 are two co-receptor PRRs for MAMP recognition [54]. To test their potential roles in PpEli2 perception, we silenced *BAK1* and *SOBIR1* individually via TRV-based VIGS in *N. benthamiana*, and then infiltrated GFP, PyolNLP7, or PpEli2 protein in these silenced plants after 3 weeks. Cell death and H_2_O_2_ accumulation triggered by PpEli2, but not PyolNLP7, were abolished in plants with the silencing of either *BAK1* or *SOBIR1* (Fig. 4A–C), which demonstrates that they are both required for the PpEli2-triggered immune response.

### REli binds to PpEli2 and co-receptors BAK1 and SOBIR1

**Figure 5:**
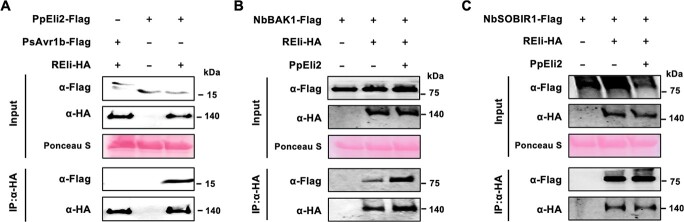
PpEli2 binds to REli and enhances the association between REli and BAK1 *in planta*. (A) PpEli2 binds to REli *in planta*. HA-tagged REli was co-expressed with Flag-tagged PpEli2 and Flag-tagged PsAvr1b in *N. benthamiana* for 48 hours. (B, C) REli binds to NbBAK1 and NbSOBIR1 *in planta*. PpEli2 enhances the association between REli and BAK1 *in planta*. HA-tagged NbREli and Flag-tagged NbBAK1 or NbSOBIR1 were co-expressed in *N. benthamiana* leaves for 48 hours. Before harvest, the leaves were treated with purified PpEli2 (1 μM) for 20 minutes. Protein complexes were immunoprecipitated with α-HA from total proteins. The bound proteins were detected by immunoblotting using α-Flag. These experiments were performed at least three times.

To test physical interaction between PpEli2 and REli, C-terminal Flag-tagged PpEli2 and HA-tagged REli were generated to perform a co-immunoprecipitation assay in *N. benthamiana*. The result showed that PpEli2 can bind REli *in planta* (Fig. 5A). We also showed that REli binds to its two co-receptors, BAK1 and SOBIR1 (Fig. 5B,C), with REli–BAK1 interaction being significantly enhanced by PpEli2 treatment (Fig. 5B). According to the results of protein–protein interaction, we speculate a model that REli recruits BAK1 and SOBIR1 to form a complex for PpEli2 perception.

### PpEli2 confers broad-spectrum resistance against filamentous pathogens in various plant species

**Figure 6:**
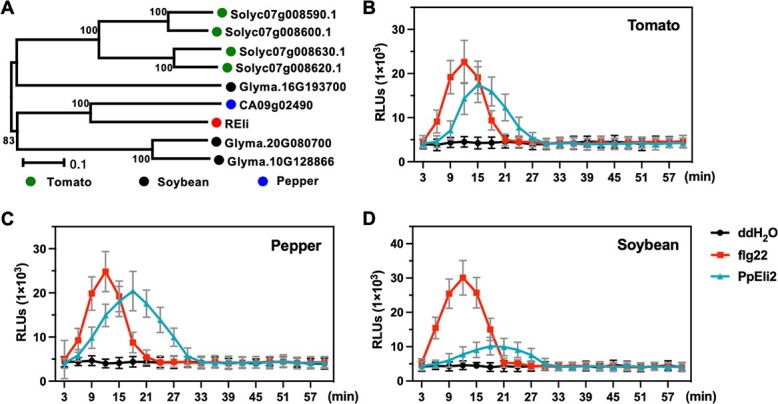
PpEli2 triggers ROS burst in various vegetable plant species*.* (A) REli is conserved in plants. The aa sequence alignment of REli and its orthologs from tomato, soybean, and pepper were used to construct the phylogenetic tree with the neighbor-joining method. (B–D) Luminol-based chemiluminescence assays showed significant ROS production in tomato (B), pepper (C), and soybean (D) leaves treated with 1 μM PpEli2. flg22 and ddH_2_O were used as the positive and negative control. Error bars represent mean ± standard deviation (*n* ≥ 9). These experiments were performed with at least three biological replicates.

**Figure 7:**
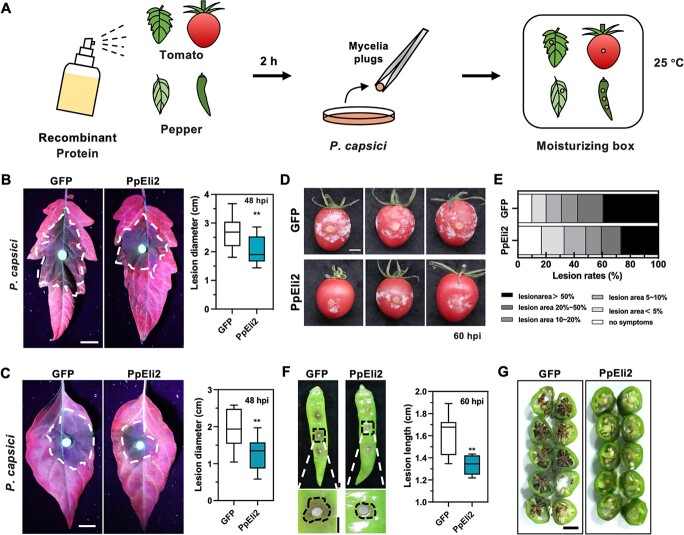
PpEli2 improves resistance to *P. capsici* in tomato and pepper. (A) Schematic diagram of *P. capsici* inoculation in tomato and pepper after recombinant protein treatment. (B, C) Disease phenotypes and lesion measurements of tomato and pepper leaves sprayed with 6 μM PpEli2 followed by *P. capsici* inoculation for 48 hpi. Scale bar = 1 cm. Error bars represent mean ± standard deviation (*n* ≥ 11). (D, F) Disease phenotypes of tomato (D) and pepper (F) fruits sprayed with PpEli2 protein followed by *P. capsici* infection for 60 hpi. (E) Statistics of lesion areas on tomato fruits sprayed with PpEli2. Error bars represent mean ± standard deviation (*n* ≥ 10). (G) Disease severity of pepper fruit rot at 72 hpi. Data were analyzed by the Shapiro–Wilk test to determine normality and log normality across groups. Groups passing the normality test were then analyzed by the unpaired *t* test (^**^, *P* < .01). These experiments were performed with five biological replicates, with similar results.

We identified REli orthologs in soybean (*Glycine max*), tomato (*Solanum lycopersicum*), and pepper (*Capsicum annuum*) via a BLASTP search (Fig. 6A). We propose that PpEli2 can improve BSR in various plant species by interacting with REli orthologs. To test this hypothesis, a luminol-based chemiluminescence assay was conducted firstly to show that PpEli2 induced a significant ROS burst in tomato, pepper, and soybean leaves (Fig. 6B–D). In China, Hang pepper and cherry tomato are important vegetables suffering substantial losses from *P. capsici* infection, and thus we assessed controlling activity of PpEli2 against the leaves and fruit rot. Tomato and pepper leaves and fruits were sprayed with 6 μM PpEli2 protein, and then inoculated with *P. capsici* mycelium plugs at 2 hours post-treatment (Fig. 7A). Compared with the negative control, PpEli2-treated leaves had reduced disease lesions (Fig. 7B and C), tomato fruits exhibited lower disease indexes (Fig. 7D and E), and pepper fruits showed smaller disease lesions and attenuated water-soaking symptoms (Fig. 7F and G).

**Figure 8:**
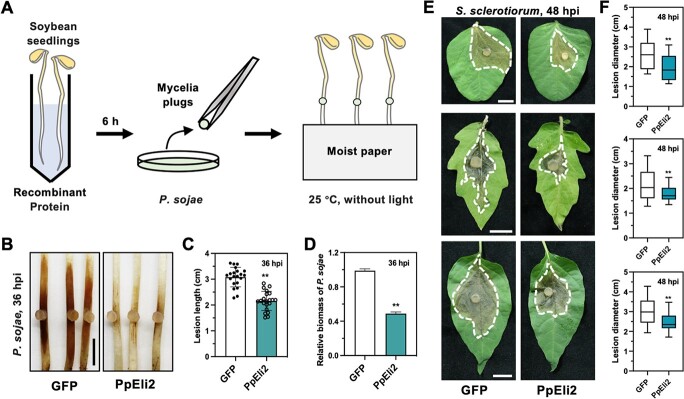
PpEli2 confers BSR against filamentous pathogens. (A) Schematic diagram of *P. sojae* inoculation of etiolated soybean seedlings after recombinant protein treatment. (B, C) Disease phenotypes and lesion length measurements of etiolated soybean seedlings treated with PpEli2 and followed by *P. sojae* inoculation at 36 hpi. Error bars represent mean ± standard deviation (*n* ≥ 10). (D) Relative biomass of *P. sojae* was determined by qPCR. Error bars represent mean ± standard deviation (*n* ≥ 9). (E) Spraying PpEli2 (6 μM) induced resistance to *S. sclerotiorum* in soybean, tomato, and pepper leaves at 48 hpi. Scale bar = 1 cm. (F) Diameters of disease lesions were measured at the indicated time points. GFP was used as a negative control. Error bars represent mean ± standard deviation (*n* ≥ 12). Data were analyzed by the Shapiro–Wilk test to determine normality and log normality across groups. Groups passing the normality test were then analyzed by the unpaired *t* test (^**^, *P* < .01). All the experiments were performed with five biological replicates.

Furthermore, we investigated the ability of PpEli2 to confer *P. sojae* resistance in soybean. Etiolated soybean seedlings were incubated in solution containing 6 μM GFP or PpEli2 for 6 hours, and subsequently challenged with *P. sojae* for 36 hours (Fig. 8A). Compared with the GFP control, pretreatment of PpEli2 significantly reduced disease lesions and *P. sojae* biomass on soybean hypocotyls (Fig. 8B–D). Together, our results demonstrate that PpEli2 can confer BSR against *Phytophthora* pathogens in various plant species. The ability of PpEli2 to confer resistance against the notorious fungal pathogen *S. sclerotiorum*, which has a broad host range that includes several important plants [55–57], was also assessed. Tomato, pepper, and soybean leaves were pretreated with either PpEli2 or GFP followed by *S. sclerotiorum* mycelium inoculation for 48 hours. PpEli2 significantly reduced leaf lesions caused by *S. sclerotiorum* in all three plants (Fig. 8E and F), suggesting that PpEli2 can also confer fungal resistance in various vegetable plants.

## Discussion

*Phytophthora* disease management is an ongoing challenge despite significant improvements in chemical pesticides and plant resistance gene breeding. *Phytophthora* is notoriously adept at overcoming these control strategies [58, 59]. Pesticide residues negatively affect food safety, human health, and the environment [60]. The long cycle time in conventional breeding limits its contribution to *Phytophthora* disease control. A bottleneck in the genetic engineering approach is the availability of promising transgenic candidates that can confer high-level BSR with minimal side effects. Given this situation, biological control, as an environment-friendly disease management method, has received remarkable attention in recent years. *P. periplocum* is potentially a promising biocontrol agent due to its aggressive parasitism on numerous plant pathogenic fungi and oomycetes [61]. In this study, we for the first time identify the elicitin-like PpEli2 as a *P. periplocum* MAMP and demonstrate its suppression effect on *Phytophthora* and fungal pathogens *in planta*.

We dissected the PpEli2-triggered plant immune pathway in detail using recombinant PpEli2 protein produced by *E. coli*. Significantly upregulated during plant–*P. periplocum* interaction, PpEli2 is directly perceived by a novel LRR-RLP, REli, which is the first elicitin receptor identified in *N. benthamiana* and is conserved in various vegetable crops. It is likely that successful PpEli2–REli interaction requires proper protein folding, which may explain the observations that PpEli2 stability and function are temperature- and pH-sensitive. REli interacts with co-receptors BAK1 and SOBIR1 to transduce immune signals, which is common for LRR-RLPs lacking the cytoplasmic kinase domain [62–64]. For example, perception of *Valsa mali* PAMP VmE02 requires its RLP receptor RE02 to form a complex with the two RLKs SOBIR1 and BAK1 [65]. Interestingly, PpEli2 significantly enhances the interaction between REli and BAK1. Downstream events of PpEli2–REli, REli–BAK1, and REli–SOBIR1 interactions include HR cell death, ROS burst, and enhanced plant resistance to various filamentous pathogens.

In the plant apoplastic space between plant–microbe interaction, the natural concentration of MAMPs is usually low, at which the molecule may not cause HR. Here, to screen and identify the objective proteins efficiently, we used the overexpression system in *N. benthamiana*, and then confirmed the phenotype with prokaryotic expression in *E. coli*. We found that PpEli2 induces HR-cell death in a concentration-dependent manner; however, a lower concentration of PpEli2 protein that failed to induce cell death still induced plant immunity, suggesting that PpEli2-induced cell death is not essential for the protective effect of PpEli2 in plants against *Phytophthora*. In addition, the higher concentrations of PpEli2 may cause side effects in the crop product. Here, we found the spray of PpEli2 protein did not cause any obvious phenotype in tomato, pepper, and soybean, and therefore the treatment of plants at a reasonable concentration will avoid adverse effects on plants.

For resistance breeding, BSR is a desirable trait as it confers resistance against more than one pathogen species or against the majority of races or strains of the same pathogen [66]. PpEli2 confers resistance to diseases caused by two *Phytophthora* species (*P. capsici* and *P. sojae*) and a fungus (*S. sclerotiorum*). The dicotyledonous crops tested include tomato, pepper, and soybean. It is likely that testing more vegetable crops and oomycetes/fungi could further expand the resistance spectrum of PpEli2 on both the plant and the pathogen side. Disease resistance in transgenic plants can be established by expressing either elicitors [67] or receptors [29]. Both the elicitin PpEli2 and the receptor REli characterized in this study are promising candidates for the development of resistant cultivars. Given the sensitivity of PpEli2 to high temperature (i.e. >37°C), its protein sequence could be engineered to improve tertiary structure stability but still retain high binding affinity to REli. This enhanced version of PpEli2 would be an ideal biocontrol agent conferring BSR in real-world field conditions.

In conclusion, our work shows that the *P. periplocum* MAMP PpEli2 significantly improves BSR to *Phytophthora* and fungal pathogens in *N. benthamiana*, tomato, pepper, and soybean. REli, a novel LRR-RLP receptor, recognizes PpEli2 and forms a complex with two co-receptors, BAK1 and SOBIR1, to trigger the downstream immune response. PpEli2 can potentially be used as a bioactive molecule to control plant diseases in an environment-friendly manner. PpEli2 induces HR and immunity in a dosage-dependent manner.

## Materials and methods

### Plasmid construction

The binary vector pBINHA was used for *Agrobacterium*-mediated transient gene expression. Genes were inserted via the SmaI site. For VIGS assays, pYL156 BamHI and EcoRI sites were used for gene cloning. For prokaryotic expression, genes were inserted into pET32a via BamHI and XhoI sites. Gene sequences used in this study are listed in Supplementary Data Table S1. Primers used in this study are listed in Supplementary Data Table S3.

### Plant growth conditions, microbial strains, and pathogen infection assays

*N. benthamiana*, tomato, pepper, and soybean were grown in chambers at 25°C, 16/8 hours light/dark [68]. The *Phytophthora* pathogens (*P. capsici* isolate LT263, *P. sojae* isolate P6497, and *P. nicotianae* isolate 025) and fungal pathogen *S. sclerotiorum* strain WMA1 were cultured at 25°C in the dark on 10% (v/v) V8 medium and potato dextrose agar medium, respectively [69].

To evaluate disease resistance in agroinfiltrated *N. benthamiana*, leaves were inoculated with *P. capsici* mycelium plugs at 24 hours after infiltration. Disease lesions were measured at 36 hours post-infiltration (hpi) [70]. Agroinfiltrated leaves were collected at 48 hours after infiltration for western blot assays. Relative biomass of *P. capsici* in infected leaves was determined by qPCR as previously described [71]. Disease resistance was also assessed after recombinant protein infiltration. Each *N. benthamiana* leaf was infiltrated with 200 nM GFP control on one half and the same concentration of PpEli2 on the other half. Methods used for *P. capsici* inoculation assay and disease development evaluation were similar to those used for transient expression in *N. benthamiana* [53, 63]*.*

In spray inoculation tests of tomato and pepper, equal amounts of recombinant protein (6 μM) were sprayed evenly on leaves and fruits. *P. capsici* mycelium plugs were inoculated into leaves 2 hours after spraying. Disease lesion diameters or lengths were measured at designated time points after inoculation. The disease index and disease scores were calculated and classified as previously described [47, 72–74]. *P. parasitica* and *S. sclerotiorum* inoculation methods were similar to that for *P. capsici*. For *P. sojae* inoculations, 4-day-old etiolated soybean seedling hypocotyls were kept in purified protein solution for 6 hours without light and then inoculated with *P. sojae* mycelium as previously described [68]. Soybean leaves from 10-day-old plants were challenged with *S. sclerotiorum* for 48 hours.

### *Agrobacterium*-mediated transient expression and VIGS in *N. benthamiana*

Leaves of 4- to 6-week-old *N. benthamiana* leaves were agroinfiltrated with *Agrobacterium* containing the constructs and the P19 silencing suppressor in a 1:1 ratio with optical density (OD) of 0.3 at 600 nm [75–77]. For VIGS assays, *Agrobacterium* cells with the pTRV1, pTRV2:BAK1, pTRV2:SOBIR1, or pTRV2:REli construct were harvested and resuspended in infiltration buffer to an OD of 0.8. A mixture of *Agrobacterium* cultures containing the pTRV1 and pTRV2 constructs was injected into two primary leaves of a four-leaf-stage plant [53, 63].

### Prokaryotic expression of recombinant proteins and stability evaluation

The coding sequence of PpEli2 was amplified and cloned into the pET32a vector. Then, pET32a–PpEli2 was transformed into *E. coli* strain BL21, Rosetta, or BL21(DE3). Protein expression was induced by 0.1 mM isopropylthio-β-galactoside (IPTG) and incubation at 16–37°C for 12 hours [78]. ImageJ software was used to quantify protein concentrations in SDS–PAGE gel bands as described previously [79]. For the evaluation of protein stability, the recombinant protein was incubated at 4–75°C for between 3 hours and 2 months before testing its activity.

### Co-immunoprecipitation assay

Proteins were extracted using HEPES extraction with protease inhibitor cocktail (Roche) [80], and then incubated with α-HA beads (Abmart) for 8–10 h. The beads were collected and then washed six times with 1 × TBS at 4°C. Proteins were released from the beads by incubating at 100°C for 5 minutes with 1 × TBS. Immunoprecipitates were separated by SDS–PAGE gels and then immunoblotted with anti-Flag (Sungene) or anti-HA (Sungene). The ECL Western Blotting Detection Kit (GE) was used to detect the blots [22, 63].

### Electrolyte leakage assay and DAB staining

*N. benthamiana* leaf disks were soaked in 5 ml of distilled water at room temperature for 2 hours. The conductivity of the bathing solution was then measured using a conductivity meter (Con 700, Consort) and calculated as previously described [81]. For DAB staining, *N. benthamiana* leaves were stained with 1 g/l DAB solution for 8 hours in the dark, and decolored with ethanol [2].

### Oxidative burst and MAPK assays

ROS production was determined enzymatically with reaction buffer in 96-well plates containing 20 μg/ml Horseradish peroxidase (HRP), 20 μM L-012 (Waco), and either 1 μM purified protein or ddH_2_O (mock). Then luminescence detection was performed using a microwell plate reader. Light emissions were measured at 3-minute intervals [82–84]. For the MAPK assay, leaves treated with 1 μM protein were extracted at 30 minutes post-treatment, then MAPK was detected with anti-phospho-p44/p42 MAPK antibody (Cell Signaling).

### Statistical analysis

GraphPad Prism 8.3.0 software was used for statistical analysis of all data. The Shapiro–Wilk test was used to determine normality and log normality across groups. Groups passing the normality test were analyzed by either one-way ANOVA with the *post hoc* Dunnett’s multiple comparisons test or the unpaired *t* test. Results are expressed as the mean ± standard deviation of replicates.

## Acknowledgements

We thank Dr Zhiyuan Yin (Nanjing Agricultural University) for kind help. This work was supported by the National Natural Science Foundation of China (32272495, 31801715, 31721004) and the Natural Science Foundation of Jiangsu Province (BK20220147).

## Author contributions

M.J. and D.D. designed this work. M.J., K.Y. and H.P. wrote the manuscript and performed data analysis. K.Y., Y.W., D.S., R.J., Y.D., Y.Z., D.L., J.L., and X.R. performed the experiments.

## Data availability

The data generated herein to support the results of this study are presented in the paper and its Supplementary Information files. Moreover, the generated and analyzed datasets of this study are available from the corresponding authors upon request.

## Conflict of interest

The authors declare that they have no competing and conflict interests.

## Supplementary data

Supplementary data is available at *Horticulture Research* online.

## Supplementary Material

Web_Material_uhac255
