# Supplementary material for: The Pythium periplocum elicitin PpEli2 confers broad-spectrum disease resistance by triggering a novel receptor-dependent immune pathway in plants
Source: Hortic Res. 2022 Nov 15;10(2):uhac255. doi: 10.1093/hr/uhac255 (PMC10390855; doi:10.1093/hr/uhac255)
Supplement: Web_Material_uhac255 [file web_material_uhac255.zip › PpEli2_Supplementary Figures_V5_R1.docx]

**
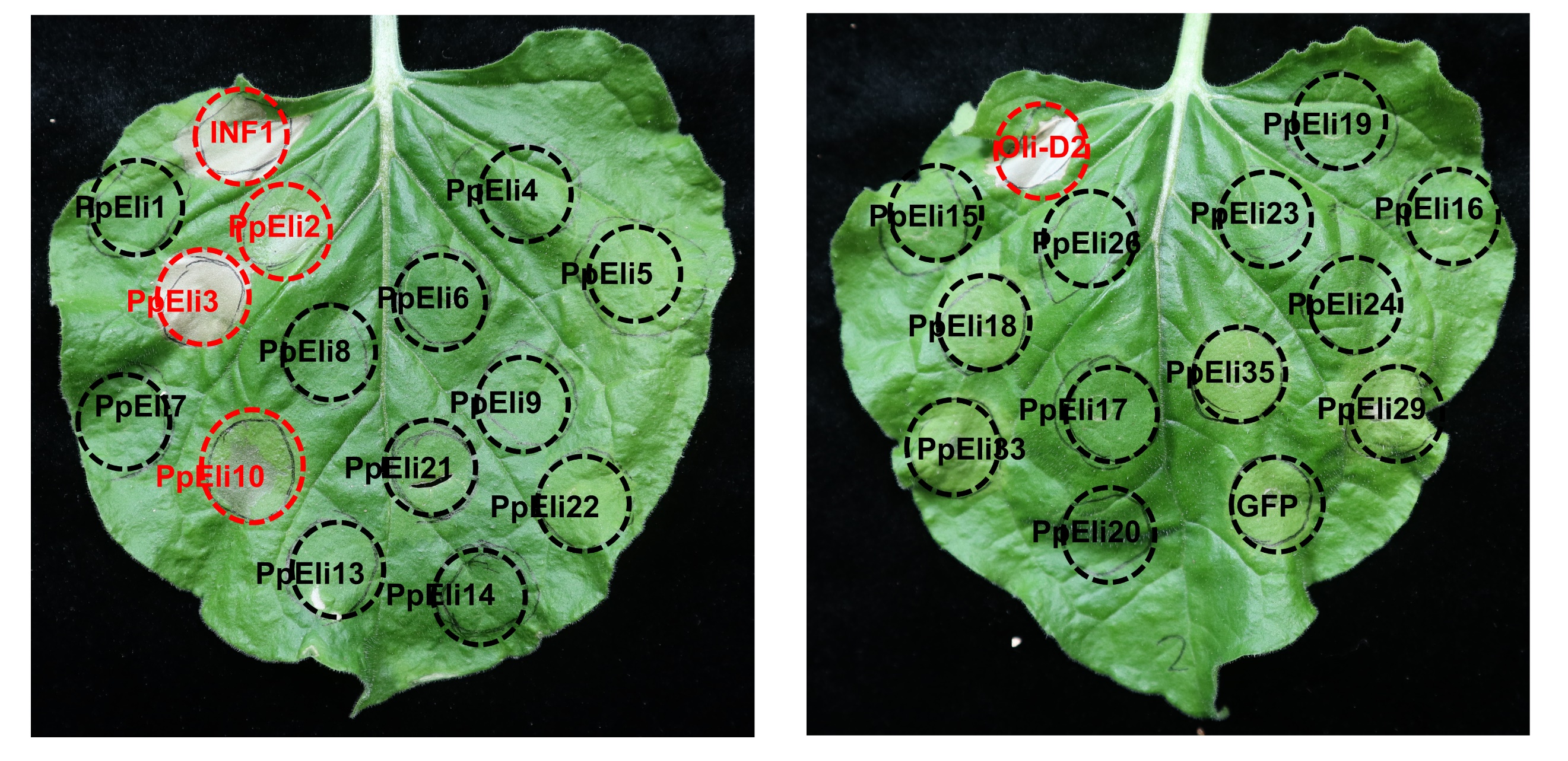
**

**Figure S1.** Screening for cell death inducing elicitins and elicitin-like proteins in *P. periplocum*. PpEli proteins, the cell death-inducing INF1/Oli-D2 (positive control), and the empty control (GFP) were transiently expressed in different areas of *N. benthamiana* leaves via agroinfiltration.


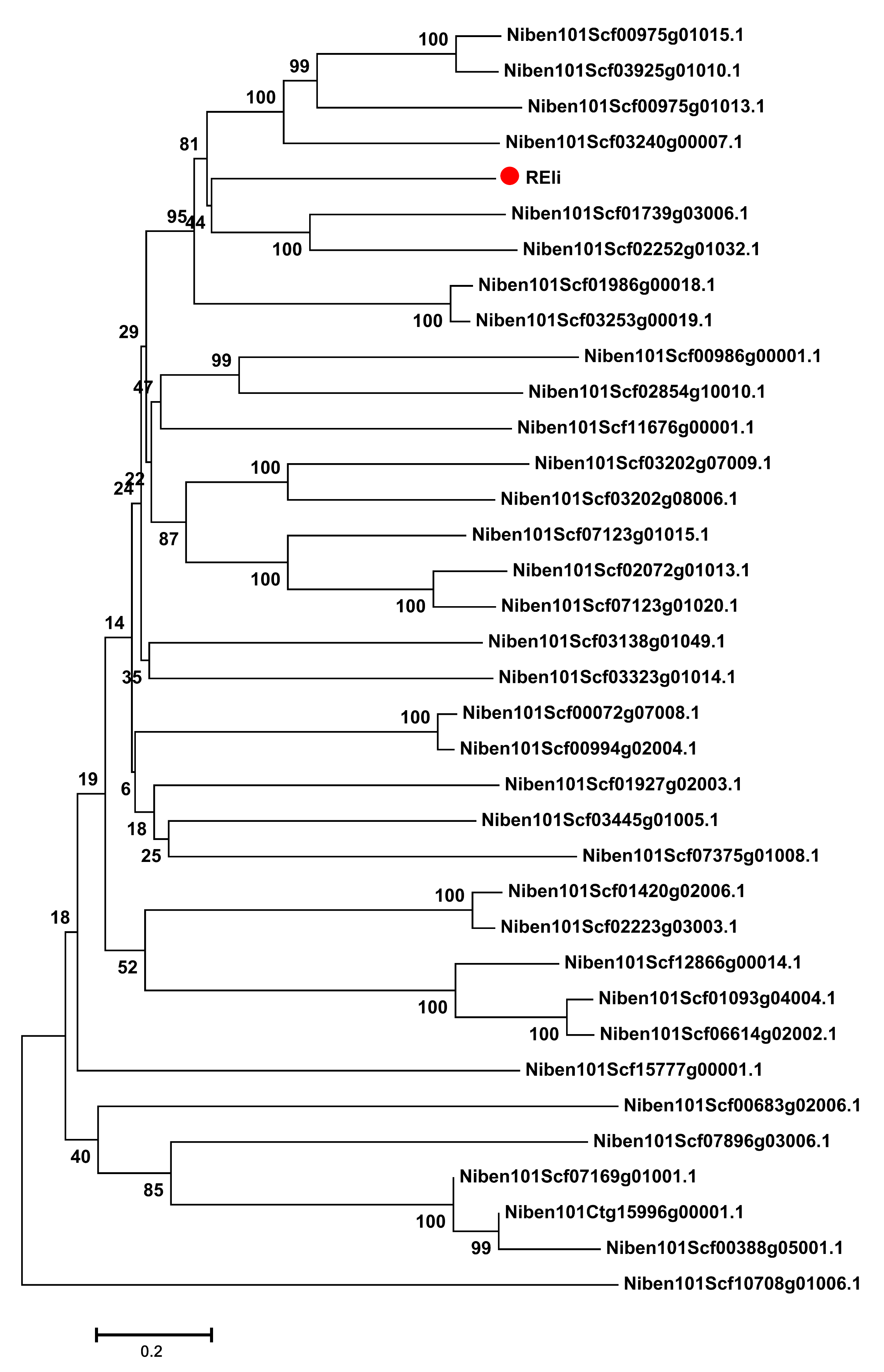


**Figure S2.** The phylogenetic tree of RLPs from *N. benthamiana*. Red cycle indicates the receptor REli that recognizes PpEli2 in this study.


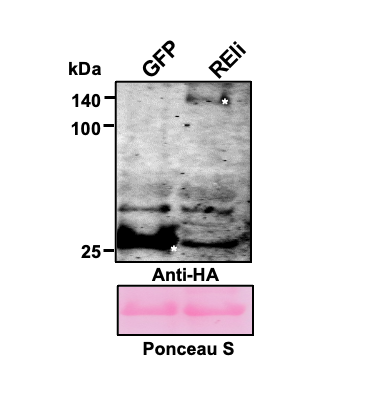


**Figure S3.** Immunoblotting analysis of REli expressed in *N. benthamiana*. Total proteins were extracted for immunoprecipitation of protein complexes using α-HA.
