# Supplementary material for: The Pythium periplocum elicitin PpEli2 confers broad-spectrum disease resistance by triggering a novel receptor-dependent immune pathway in plants
Source: Hortic Res. 2022 Nov 15;10(2):uhac255. doi: 10.1093/hr/uhac255 (PMC10390855; doi:10.1093/hr/uhac255)
Supplement: Web_Material_uhac255 [file web_material_uhac255.zip › PpEli2_Supplementary Table 2_V5.docx]

| **Temperature** | | 16 °C | | | 25 °C | | | 37 °C | | |
| --- | --- | --- | --- | --- | --- | --- | --- | --- | --- | --- |
| **OD** | | 0.4-0.6 | 0.6-0.8 | 0.8-1.0 | 0.4-0.6 | 0.6-0.8 | 0.8-1.0 | 0.4-0.6 | 0.6-0.8 | 0.8-1.0 |
| ***E. coli* stains** | BL21 | + | + | + | + | + | - | - | - | - |
|  | Rosetta | ++ | ++ | + | + | + | + | + | + | + |
|  | BL21(DE3) | +++ | ++ | ++ | ++ | ++ | + | + | + | + |
| Production: -, no; +, low; ++, medium; +++, high | | | | | | | | | | |

**Table S2. Evaluation of expression conditions and stability of Recombinant protein PpEli2.**

Evaluation of expression conditions of PpEli2 recombinant protein. Recombinant constructs were transformed into *E. coli* strain BL21, Rosetta or BL21(DE3). Expression of 6xHis-tagged PpEli2 was induced by adding 0.1mM IPTG and incubating at 16°C, 25°C or 37 °C for 12 h. (-: no; +: low; ++: medium; +++: high).
